# Cardioprotection against Ischemia/Reperfusion by Licochalcone B in Isolated Rat Hearts

**DOI:** 10.1155/2014/134862

**Published:** 2014-08-21

**Authors:** Jichun Han, Dong Wang, Bacui Yu, Yanming Wang, Huanhuan Ren, Bo Zhang, Yonghua Wang, Qiusheng Zheng

**Affiliations:** ^1^Key Laboratory of Xinjiang Endemic Phytomedicine Resources, Pharmacy School, Shihezi University, Ministry of Education, Shihezi 832002, China; ^2^Qianfoshan Hospital of Shandong Province, Jinan 250014, China; ^3^Binzhou Medical University, Yantai 264003, China

## Abstract

The generation of reactive oxygen species (ROS) is a major cause of heart injury induced by ischemia-reperfusion. The left ventricular developed pressure (LVDP) and the maximum up/down rate of left ventricular pressure (±*dp*/*dt*_max⁡_) were documented by a physiological recorder. Myocardial infarct size was estimated macroscopically using 2,3,5-triphenyltetrazolium chloride staining. Coronary effluent was analyzed for lactate dehydrogenase (LDH) and creatine kinase (CK) release to assess the degree of cardiac injury. The levels of C-reactive protein (CRP), interleukin-8 (IL-8), tumor necrosis factor-*α* (TNF-*α*), and interleukin-6 (IL-6) were analyzed to determine the inflammation status of the myocardial tissue. Cardiomyocyte apoptosis analysis was performed using the In Situ Cell Death Detection Kit, POD. Accordingly, licochalcone B pretreatment improved the heart rate (HR), increased LVDP, and decreased CK and LDH levels in coronary flow. SOD level and GSH/GSSG ratio increased, whereas the levels of MDA, TNF-*α*, and CRP and activities of IL-8 and IL-6 decreased in licochalcone B-treated groups. The infarct size and cell apoptosis in hearts from licochalcone B-treated group were lower than those in hearts from the I/R control group. Therefore, the cardioprotective effects of licochalcone B may be attributed to its antioxidant, antiapoptotic, and anti-inflammatory activities.

## 1. Introduction

Ischemic heart disease is a leading cause of death worldwide and is the most common consequence of coronary artery disease. Reperfusion of an occluded human coronary can effectively reduce overall mortality; the restoration of blood flow through the previously ischemic myocardium can elevate reperfusion injury symptoms, including cardiomyocyte dysfunction and cell death [1]. Previous studies showed that the myocardial response to ischemia-reperfusion can be manipulated to delay injury. Thus, studies on the mechanisms of cardioprotection were conducted. Various interventions that target the preischemic period and/or reperfusion have been investigated for their efficacy in preventing myocardial injury after ischemic insult. Among the most effective experimental strategies are interventions such as ischemic preconditioning and its pharmacological mimicking [2].

Myocardial ischemia-reperfusion injuries in pathological disorders include reperfusion arrhythmias, transient mechanical dysfunction, myocardial stunning, and cell death [3]. Oxidative stress caused by reactive oxygen species (ROS) has a considerable role in ischemia/reperfusion (I/R) injury, which impairs cardiac function [4]. Ischemia/reperfusion leads to an imbalance between antioxidants and the accumulation of toxic free radicals, which increase the susceptibility of tissues to oxidative damage via lipid peroxidation, protein oxidation, and DNA oxidation [5]. The cardioprotective effects of various antioxidants have been studied in vivo and in vitro using hearts [6]. Several studies revealed that the addition of antioxidants or scavengers, such as superoxide dismutase (SOD) and catalase, could reduce infarct size [7–10].

Flavonoids have unique antioxidant properties and other pharmacological activities that may be relevant to protecting the heart from ischemia-reperfusion injury. These flavonoids may prevent production of oxidants (e.g., by inhibition of xanthine oxidase and chelation of transition metals), may inhibit oxidants from attacking cellular targets (e.g., by electron donation and scavenging activities), may block propagation of oxidative reactions (e.g., by chain-breaking antioxidant activity), and may reinforce cellular antioxidant capacity (e.g., by minimizing the effects of oxidants on antioxidants and by inducing expression of endogenous antioxidants). Flavonoids also possess anti-inflammatory and antiplatelet aggregation effects by inhibiting relevant enzymes and signaling pathways. Such activities ultimately lower oxidant production and enhance the reestablishment of blood in the ischemic zone. Finally, flavonoids exhibit vasodilatory effects through a variety of mechanisms, one of which may be the interaction with ion channels. These multifaceted activities of flavonoids corroborate their use as potential therapeutic intervention tools to ameliorate ischemia-reperfusion injury [11].

Licochalcone B, which belongs to the retrochalcone family, is isolated from the roots of Chinese licorice. Studies on the biological activities of licochalcone B are in the initial stage. Licochalcone B showed high antioxidant and free radical-scavenging activities [12, 13]. Experimental studies suggested that licochalcone B has several other useful pharmacological properties, such as anti-inflammatory activities [14], leukotriene inhibition action [15], and preventive activity against glucose-mediated protein damage [16]. We aimed to evaluate the cardioprotective effects of licochalcone B and mechanisms underlying such effects.

## 2. Materials and Methods

### 2.1. Animals

Adult male SD rats, weighing 250 g to 300 g, were obtained from Xinjiang Medicine University Medical Laboratory Animal Center (SDXK 2011-004) and housed in a room at 22°C to 25°C, 50% to 60% relative humidity, and a 12 h light/12 h dark cycle. All experimental procedures were approved by the Institutional Animal Care and Use Committee of National Institute Pharmaceutical Education and Research.

### 2.2. Test Compounds, Chemicals, and Reagents

Licochalcone B (purity ≥ 98%) was purchased from Shanghai Li Chen Biotechnology Co., Ltd. (Shanghai, China). 1,1,3,3-Tetramethoxypropane was obtained from Fluka Chemical Co. (Ronkonkoma, NY). 2,3,5-Triphenyltetrazolium chloride (TTC), oxidized glutathione, and reduced glutathione were purchased from Sigma Chemical Co. (St. Louis, MO). Other chemicals and reagents were of analytical grade.

### 2.3. Drug Administration and Surgical Procedure

The rats were randomly divided into seven groups as follows: control (Sham), I/R, and licochalcone B (0.5, 1, 3, and 5 *μ*g/mL). Hearts in the control group were perfused during the 90 min stabilization period. Hearts from the IR group were subjected to 20 min of zero-flow global ischemia and 45 min of reperfusion after stabilization. Hearts in licochalcone B treatment group were stabilized for 30 min and treated with Krebs-Henseleit (K-H) buffer solution containing licochalcone B (0.5, 1, 3, and 5 *μ*g/mL). Global ischemia and reperfusion were established for 45 min.

### 2.4. IR

Rats were anesthetized with chloral hydrate (350 mg/kg). The hearts were excised quickly and immediately immersed in ice-cold K-H buffer (pH 7.4) containing 118 mM NaCl, 1.2 mM KH_2_PO_4_, 4.7 mM KCl, 1.7 mM CaCl_2_, 1.2 mM MgSO_4_, 20 mM sodium acetate, and 10 mM glucose. The buffer was maintained at a temperature of 37°C and infused continuously with oxygen. The excised hearts were cannulated through the aorta by a Langendorff apparatus and were perfused in retrograde with K-H buffer containing 95% O_2_ and 5% CO_2_ throughout the experiment. Perfusion pressure was maintained at 75 mmHg. A water-filled latex balloon coupled to a pressure transducer (Statham) was inserted into the left ventricular cavity via the left auricle for pressure recording. Ventricular end-diastolic pressure (VEDP) was adjusted between 5 and 12 mmHg. The hearts were stabilized for 30 min, after which global ischemia and reperfusion were established for 15 and 45 min, respectively. The hearts from the control group were perfused and subjected to a 90 min stabilization procedure. During the experiment, left ventricular developed pressure (LVDP), LVEDP, heart rate (HR), and rate of developed pressure during contraction and relaxation (±*dp*/*dt*_max⁡_) were monitored continuously using a 4S AD Instruments biology polygraph. The heart effluents were collected at 1 min intervals at selected times to determine coronary flow.

### 2.5. Measurement of Cellular Injury

Lactate dehydrogenase (LDH) and creatine kinase (CK) release is measured to evaluate the presence of necrotic cell death [15]. At the end of the experiment, levels of LDH and CK in the perfusate were determined spectrophotometrically via cytotoxicity detection LDH and CK kits (Nanjing Jiancheng Biological Product, Nanjing, China).

### 2.6. Evaluation of Myocardial Infarct Size

The artery was occluded for 20 min and reperfused for 45 min before the end of the experiment. These durations of ischemia and reperfusion have been successfully used in the same experimental model. To evaluate tissue death, the hearts were removed and washed in phosphate buffered saline, frozen and stored at −20°C for 30 min, and sliced into 1 mm sections perpendicularly along the long axis from apex to base. The slices were incubated in 1% TTC in pH 7.4 buffer at 37°C for 10 min to 15 min, fixed in 10% formaldehyde solution, and photographed with a digital camera to distinguish the red-stained viable and the white-unstained necrotic tissues. Areas stained in red and white were measured using an Image-Pro Plus 7.0 (Media Cybernetics, Wyoming, USA). The infarction size percentage was calculated by the following equation:

(1) $$\begin{array}{c} \%\text{Infarct}\;\text{volume}=\frac{\text{Infarct}\;\text{volume}}{\text{Total}\;\text{volume}\;\text{of}\;\text{slice}\;}\times \;100. \end{array}$$

### 2.7. Assay of Oxidative Stress

At the end of the perfusion treatments, the hearts were harvested and maintained at −70°C for subsequent analysis. The frozen ventricles were crushed to a powder using liquid nitrogen-chilled tissue pulverizer. For tissue analyses, the weighed amount of the frozen tissues was homogenized in appropriate buffer using microcentrifuge tube homogenizer.

The SOD, malondiadehyde (MDA), and glutathione/glutathione disulfide (GSG/GSSH) concentrations were analyzed spectrophotometrically according to the instruction of the assay kits (Nanjing Jiancheng Bioengineering Institute, Nanjing, China).

### 2.8. Inflammation Assay

TNF-*α*, CRP, IL-8, and IL-6 were analyzed spectrophotometrically according to the instruction of the Rat Tumor Necrosis Factor Alpha ELISA Kit, Rat C-reactive protein ELISA Kit, Rat Interleukin 8 ELISA Kit, and Rat Interleukin 6 ELISA Kit (Tsz Biosciences, Greater Boston, USA).

### 2.9. General Histology

The rat's heart was fixed in 10% formaldehyde and preserved at normal temperature. The heart was observed under an optical microscope after HE coloration. A small piece (2 mm × 1 mm × 1 mm) of subendocardial myocardium from the root of left ventricular papillary muscle was obtained and fixed in 0.1 mmol/L phosphate buffer (pH 7.2), which included 3% glutaraldehyde and 1.5% paraformaldehyde at 4°C. The piece was cut into small pieces of 1 mm^3^ and subsequently fixed in the abovementioned solution for 4 h. Moreover, the piece was fixed in 1% osmic acid again at 4°C for 1.5 h after being rinsed with phosphate buffer. Afterwards, the tissue was dehydrated by alcohol followed by dimethylbenzene and embedded in epoxy resin 618. The tissue was located by semithin sectioning and sliced into ultrathin sections (60 nm). The sections were dyed with uranium acetate and lead citrate and observed under an optical microscope.

### 2.10. Terminal Deoxynucleotidyl Transfer-Mediated dUTP Nick End-Labeling (TUNEL) Staining

We conducted TUNEL by using an In Situ Cell Death Detection Kit, POD (Roche, Germany), according to the manufacturer's instructions. After deparaffinization and rehydration, the sections were treated with protease K at 10 mmol/L concentration for 15 min. The slides were immersed in TUNEL reaction mixture for 60 min at 37°C in a humidified atmosphere in the dark. Converter-POD was used to incubate the slides for 30 min to show blue nuclear staining. The slides were analyzed by optical microscopy. The TUNEL index (%) was the ratio of the number of TUNEL-positive cells divided by the total number of cells and was used to evaluate the apoptosis index of the heart TUNEL-stained tissues. For each sample, eight randomly selected areas of TUNEL-stained slices were counted, and the average value was calculated.

### 2.11. Statistical Analysis

Data were presentedas means ± SD from at least three independent experiments and evaluated by analysis of variance (ANOVA) and followed by Student's* t*-test. The values of *P* < 0.05 were considered statistically significant. The analyses were performed using the Statistical Program for Social Sciences Software (IBM SPASS, International Business Machines Corporation, Armonk City, New York, USA).

## 3. Results

### 3.1. Licochalcone B Enhanced the Recovery of I/R-Altered Cardiac Function

We evaluated cardiac function by monitoring hemodynamic parameters, which are important indices of cardiac function. The doses of licochalcone B used in the experiments were determined during the preliminary experiments. Licochalcone B concentration of 0.5, 1, 3, or 5 *μ*g/mL was selected. The hemodynamic parameters were continuously monitored using a computer-based data acquisition system (PC PowerLab with Chart 5 software, 4S AD Instruments). The effects of licochalcone B treatment on LVDP, ±*dp*/*dt*_max⁡_, CF, and HR during I/R in the control, I/R, and licochalcone B-treated hearts are shown in Table 1. Compared with the unprotected I/R hearts, licochalcone B significantly improved functional recovery during early reperfusion, and 1 *μ*g/mL of licochalcone B group significantly improved LVEDP, ±*dp*/*dt*_max⁡_, and HR (*P < 0.05).

### 3.2. Licochalcone B Attenuated I/R-Induced Enzyme Release in Rat's Heart

To evaluate the degree of myocardial injury, we measured the release of LDH and CK. This method has been used in previous studies to evaluate the presence of necrotic cell death. Prior to ischemia, LDH and CK levels in the effluents from the control, I/R, and licochalcone B-treated groups were fundamentally similar. After 20 min of ischemia followed by 20 and 45 min of reperfusion, the leakage of CK and LDH notably increased in the I/R group compared with the control (Table 2). Pretreatment with licochalcone B at 0.5 and 1 *μ*g/mL significantly reduced the I/R-induced increase in LDH and CK release in rat's heart (**P < 0.01).

### 3.3. Licochalcone B Reduced I/R-Induced Infarct Size

**Figure 1:**
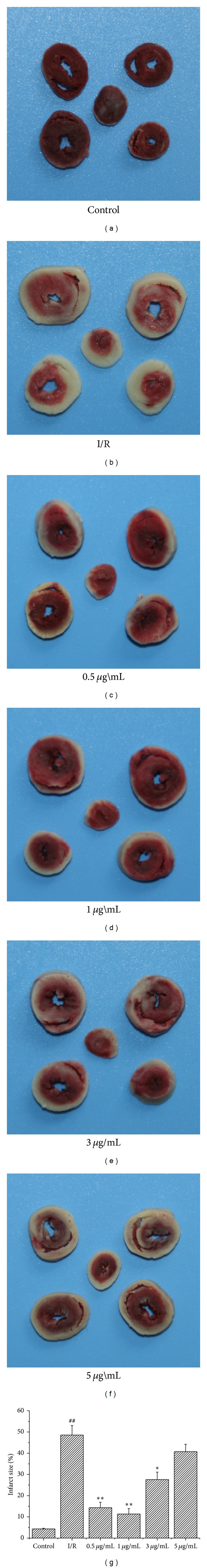
Effect of licochalcone B on the reduction of I/R-induced infarct size. ^##^ *P* < 0.01 compared with control group; *P < 0.05 and **P < 0.01 compared with the I/R group.

Myocardial infarct size can be an indicator of myocardial injury. I/R group hearts subjected to global myocardial ischemia for 20 min followed by 45 min of reperfusion showed a significant increase of risk area infarct (48.55% ± 4.47%). By contrast, licochalcone B preconditioning reduced the percentage of the I/R-induced myocardial infarct size (Figure 1). Licochalcone B preconditioning at 0.5 and 1 *μ*g/mL reduced the I/R-induced percentage of myocardial infarct size (14.34 ± 2.56% and 11.36 ± 2.53%, resp.).

### 3.4. Licochalcone B Alleviated Oxidative Stress Induced by I/R

**Figure 2:**
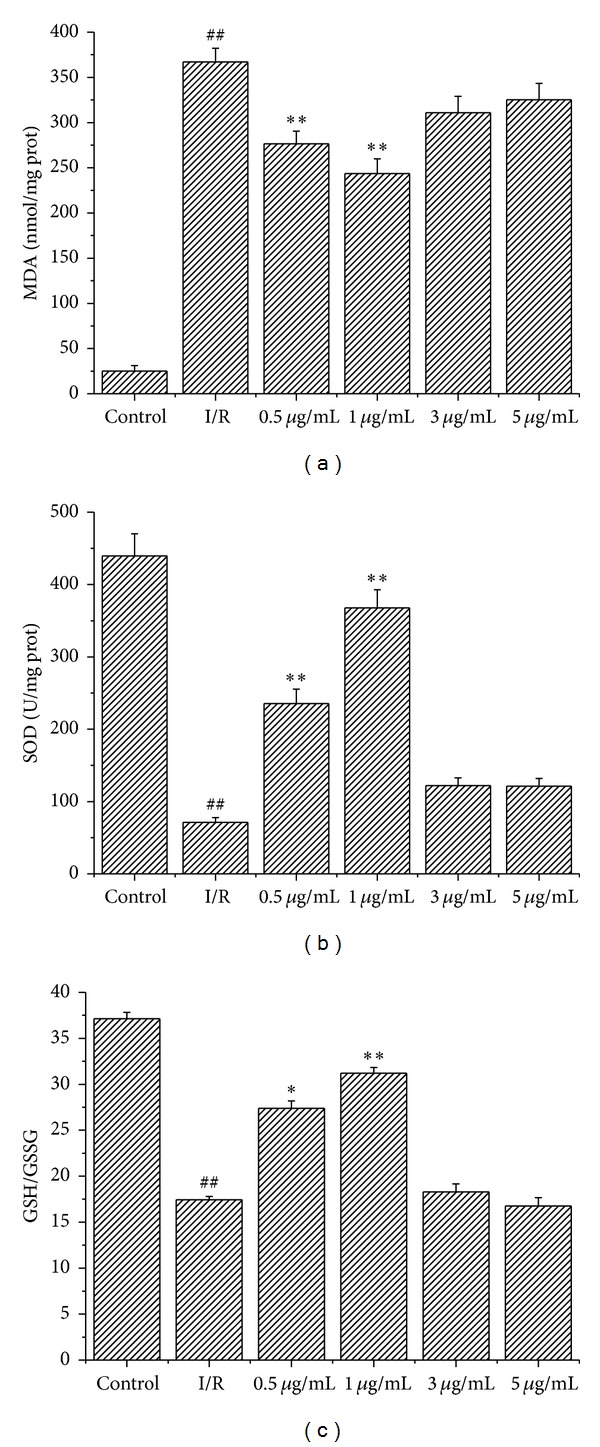
Effect of licochalcone B on cardiac contents of MDA, SOD, and GSH/GSSG in rats subjected to I/R (values are means with their standard deviation, *n* = 8). ^##^ *P* < 0.01 compared with control group; *P < 0.05 and **P < 0.01 compared with the I/R group.

ROS generation is a major factor in I/R injury. SOD, MDA, and the ratio of GSH/GSSG are indicators of oxidation. The SOD activity, MDA level, and ratio of GSH/GSSG were determined in myocardial tissue to identify the possible mechanisms underlying the cardioprotective effects of licochalcone B. MDA level significantly decreased (Figure 2(a)), whereas SOD activity (Figure 2(b)) and GSH/GSSG ratio (Figure 2(c)) significantly increased in the group pretreated with 1 *μ*g/mL licochalcone B compared with the I/R group. The 5 *μ*g/mL licochalcone B pretreatment group showed no significant difference compared with the I/R group.

### 3.5. Licochalcone B Reduced Myocardial Structure Injury Induced by I/R

**Figure 3:**
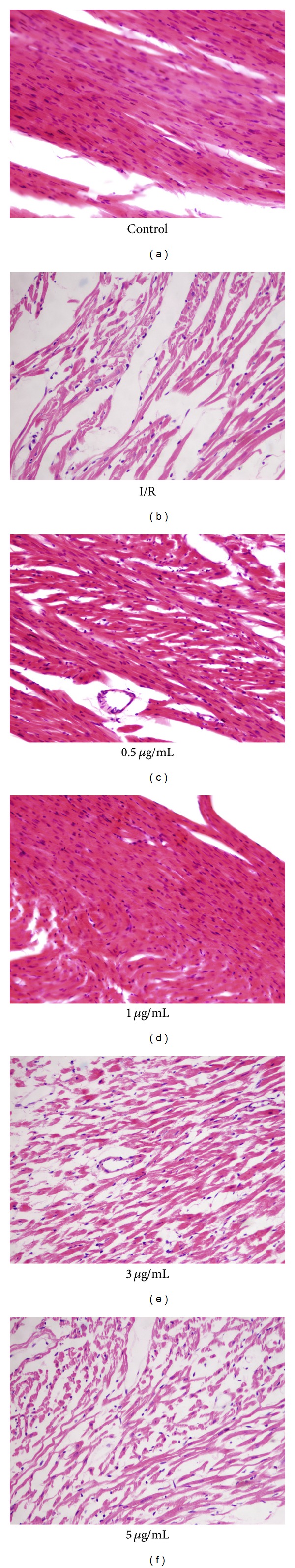
Effects of licochalcone B on cell morphology and hematoxylin and eosin (HE) staining (×200).

**Figure 4:**
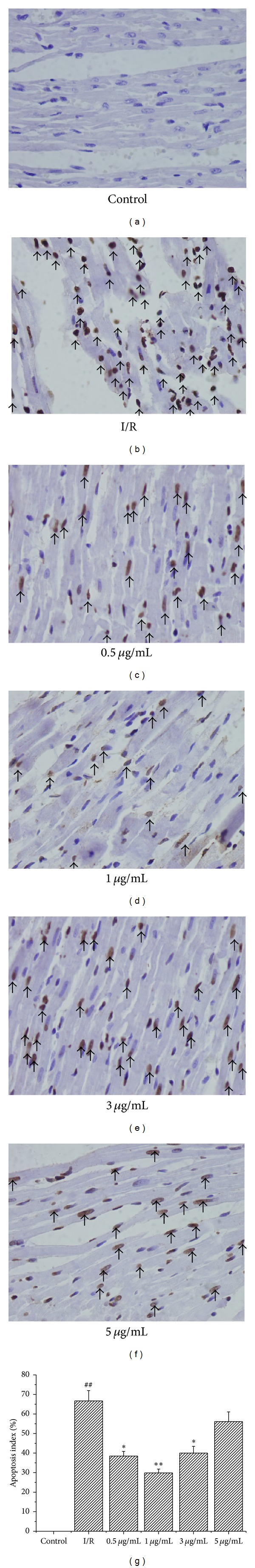
Effects of licochalcone B suppression on cardiomyocyte apoptosis (×400). Arrows indicate the apoptosis cardiomyocyte nucleus. ^##^ *P* < 0.01 compared with control group; *P < 0.05 and **P < 0.01 compared with the I/R group.

**Figure 5:**
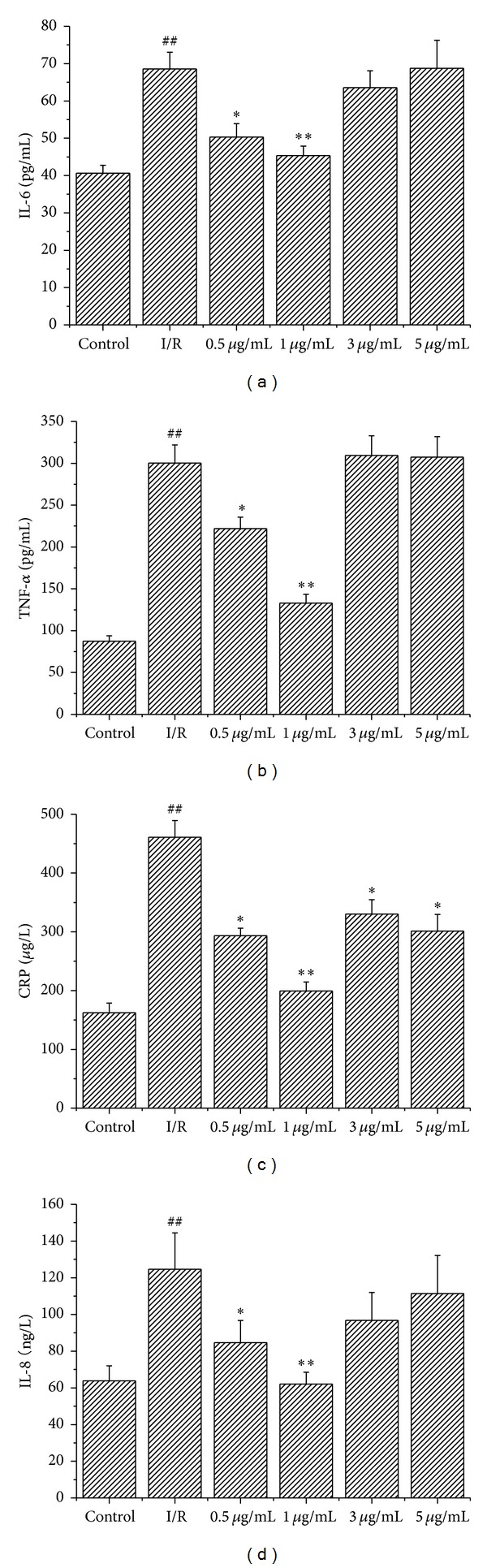
Effect of licochalcone B on cardiac composition of IL-6 and TNF-*α* in rats subjected to I/R (values are means with their standard deviation, *n* = 8). ^##^ *P* < 0.01 compared with control group; *P < 0.05 and **P < 0.01 compared with the I/R group.

The changes in the morphological structure of myocardial tissue were elevated by HE coloration. The optical microscopy of rat myocardial structure is shown in Figure 3. The myocardial structures of the control group (Figure 3(a)) were as follows: muscle fibers were neatly arranged; interstitial substance contained no edema; muscle membrane was not damaged; and muscle fibers showed no fracture, degeneration, and necrosis. By contrast, the myocardial structures of I/R group (Figure 3(b)) were as follows: muscle fibers were irregularly arranged; interstitial substance exhibited edema; muscle membrane was damaged; and muscle fibers showed fracture, degeneration, and necrosis. Compared with the I/R group, the group pretreated with licochalcone B at 0.5 (Figure 3(c)) and 1 *μ*g/mL (Figure 3(d)) showed significantly reduced I/R-induced myocardial structure injury. However, the groups pretreated with licochalcone B at 3 (Figure 4(e)) and 5 *μ*g/mL (Figure 4(f)) indicated no significant difference compared with the I/R group.

### 3.6. Licochalcone B Reduced Cardiomyocyte Apoptosis Induced by I/R

The results of ischemic reperfusion myocardium in cardiomyocyte apoptosis were evident in the section. We performed TUNEL staining to observe cardiomyocyte apoptosis. Under optical microscopy, TUNEL staining showed the absence of apoptosis in the control group (Figure 4(a)). The number of apoptotic cells increased dramatically in the I/R group (Figure 4(b)), whereas the groups pretreated with licochalcone B at 0.5 (Figure 4(c)) and 1 *μ*g/mL (Figure 4(d)) showed an obviously reduced number of apoptotic cells. The groups pretreated with licochalcone B at 3 (Figure 4(e)) and 5 *μ*g/mL (Figure 4(f)) showed no significant difference compared with the I/R group. The apoptosis percentage is shown in Figure 4(g).

### 3.7. Licochalcone B Reduced Inflammation Induced by I/R

Inflammation is an important mechanism underlying myocardial I/R injury. The presence of inflammatory cytokines (IL-6, CRP, IL-8, and TNF-*α*) associated with I/R was determined in myocardial tissue to identify the possible mechanisms underlying the cardioprotective activity of licochalcone B. The levels of IL-6 and CRP and the activities of IL-8 and TNF-*α* were determined. The content of IL-6 in the group pretreated with 1 *μ*g/mL licochalcone B (45.36 ± 2.53 pg/mL) was significantly lower (*P* < 0.01) than that in the I/R group (68.55 ± 4.47 pg/mL) (Figure 5(a)). The activity of TNF-*α* decreased from 300.24 ± 21.58 pg/mL in the I/R group to 132.97 ± 10.45 pg/mL in the group pretreated with 1 *μ*g/mL licochalcone B (*P* < 0.01) (Figure 5(b)). Compared with I/R group (461.12 *μ*g/L ± 28.10 *μ*g/L), CRP level decreased significantly in the group treated with 1 *μ*g/mL licochalcone B (199.47 pg/mL ± 15.08 pg/mL) (*P* < 0.01) (Figure 5(c)). The activity of IL-8 decreased from 124.61 ± 19.82 ng/L in the I/R group to 62.04 ± 6.49 ng/L in the group treated with 1 *μ*g/mL licochalcone B (*P* < 0.01) (Figure 5(d)).

The levels of IL-6 and IL-8 activities and CRP level and TNF-*α* activity in the group treated with 0.5 *μ*g/mL licochalcone B decreased significantly compared with the I/R group (*P* < 0.05). No significant difference was indicated between the group treated with high doses of licochalcone B (3 and 5 *μ*g/mL) and the I/R group.

## 4. Discussion

We observed the following: (1) the middle-dose licochalcone B (0.5 and 1 *μ*g/mL) pretreatment reduced I/R injury; (2) middle-dose licochalcone B suppressed the I/R-induced increase in MDA level and decrease in SOD activity and GSH/GSSG ratio; (3) middle-dose licochalcone B reduced cardiomyocyte apoptosis induced by I/R; and (4) high-dose licochalcone B (3 and 5 *μ*g/mL) indicated no cardioprotective effects. Thus, the cardioprotective effects of licochalcone B may be attributed to its antioxidant, antiapoptotic, and anti-inflammatory activities.

Studies have suggested that ROS generation is among the major factors in I/R injury [17, 18]. Under normal conditions, ROS concentration can be reduced by antioxidant systems, including antioxidant enzymes, such as SOD, and antioxidant molecules, such as GSH [19]. However, when the amount of ROS is beyond the capacity of the abovementioned enzymes and cannot be diminished in reperfusion, oxidative stress occurs. Several studies demonstrate that ROS produced in the reperfused myocardium causes oxidative stress mediated injury under antioxidant protection [11, 20]. Therefore, reducing oxidative stress is an advantageous strategy to alleviate I/R injury. Flavonoids have long been acknowledged for their unique antioxidant properties [21]. Licochalcone B is widely recognized as a major active chemical component isolated from licorice and possesses versatile biological activation, such as antioxidant and anti-inflammatory mechanisms [22]. The present results show that licochalcone B protected the tissues against myocardial I/R-induced injury. Licochalcone B treatment attenuated MDA production and enhanced SOD activity and GSH/GSSG ratio. Therefore, one of the mechanisms of the cardioprotection of licochalcone B was associated with its antioxidant effects. Licochalcone B pretreatment resulted in the attenuation of IL-6 activity and TNF-*α* production, thereby indicating that one of the mechanisms of the cardioprotection of licochalcone B was associated with its anti-inflammatory effects.

Reperfusion of the ischemic myocardium results in cardiomyocyte apoptosis and heart dysfunction [23, 24]. We observed significant myocardial dysfunction, including changes in hemodynamic parameters (LVDP, ±*dp*/*dt*_max⁡_, CF, and HR), release of enzymes (CK and LDH), and induced myocardial infarct after reperfusion of the ischemic myocardium. We also observed significant cardiomyocyte apoptosis. These phenomena are in agreement with results of numerous reports indicating that reperfusion is a key initiator of myocardial dysfunction and cardiomyocyte apoptosis associated with I/R injury. Licochalcone B significantly improved the recovery of I/R-altered hemodynamic parameters (LVDP, ±*dp*/*dt*_max⁡_, CF, and HR), decreased I/R-induced enzyme release (CK and LDH) and cardiomyocyte apoptosis rate, and attenuated infarct size.

Inflammation is involved in I/R injury. CRP, IL-8, IL-6, and TNF-*α* are proinflammatory cytokines that function in the inflammatory system [25–29]. To investigate the relationship between anti-inflammatory and the cardioprotective effects of licochalcone B, an experiment was performed to examine whether licochalcone B affected the changes in CRP, IL-8, IL-6, and TNF-*α* induced by I/R. I/R increased CRP, IL-8, IL-6, and TNF-*α* production, whereas licochalcone B treatment reduced the concentrations of these cytokines. Therefore, the suppressed infiltration of inflammatory cytokines by licochalcone B treatment may contribute to the cardioprotective effects of this flavonoid after reperfusion. Overall, licochalcone B can potentially inhibit myocardial I/R injury via anti-inflammatory activities.

Licochalcone B exhibited significant cardioprotective effects during I/R injury. These effects included the decrease in infarct volume, thereby preventing cell apoptosis. Licochalcone B decreased LDH and CK release and functioned as an anti-inflammatory agent. Licochalcone B increased the capacity of antioxygen free radical. Thus, we hypothesize that licochalcone B deserves additional experimental and clinical research in the cardiovascular milieu.

## Figures and Tables

**Table 1:** Effect of licochalcone B on cardiac function in rats subjected to I/R (values are means with their standard deviation, *n* = 8).

Physical index	Reperfusion (%)		
	15 min	30 min	45 min
LVDP			
Control	96.31 ± 3.27	94.45 ± 4.12	93.67 ± 4.92
I/R	40.50 ± 4.59^##^	47.18 ± 4.08^##^	48.76 ± 5.88^##^
0.5 *μ*g/mL licochalcone B	79.76 ± 2.06∗∗	78.28 ± 3.79∗∗	74.13 ± 5.39∗∗
1 *μ*g/mL licochalcone B	85.39 ± 2.73∗∗	82.23 ± 2.83∗∗	81.49 ± 3.41∗∗
3 *μ*g/mL licochalcone B	63.88 ± 5.54∗	57.46 ± 3.99∗	55.16 ± 2.53∗
5 *μ*g/mL licochalcone B	58.54 ± 3.97	52.96 ± 3.82	51.10 ± 4.28
+*dp*/*dt*_max⁡_			
Control	117.68 ± 2.59	116.79 ± 3.63	113.03 ± 3.71
I/R	42.26 ± 3.27^##^	52.83 ± 3.41^##^	52.62 ± 3.92^##^
0.5 *μ*g/mL licochalcone B	68.48 ± 4.84∗	75.29 ± 7.46∗	74.59 ± 4.40∗
1 *μ*g/mL licochalcone B	95.11 ± 3.50∗∗	95.76 ± 1.84∗∗	92.27 ± 2.94∗∗
3 *μ*g/mL licochalcone B	61.05 ± 4.83∗	60.36 ± 4.50∗	57.68 ± 3.66∗
5 *μ*g/mL licochalcone B	55.90 ± 3.60∗	55.09 ± 2.10∗	54.22 ± 3.97∗
−*dp*/*dt*_max⁡_			
Control	102.69 ± 3.72	99.30 ± 4.93	97.35 ± 5.01
I/R	49.22 ± 5.88^##^	56.47 ± 3.28^##^	56.05 ± 4.49^##^
0.5 *μ*g/mL licochalcone B	72.96 ± 5.38∗	74.12 ± 2.42∗	66.78 ± 5.68∗
1 *μ*g/mL licochalcone B	85.60 ± 2.40∗∗	83.62 ± 5.14∗∗	84.76 ± 7.00∗∗
3 *μ*g/mL licochalcone B	54.59 ± 6.85∗	53.50 ± 5.25∗	50.12 ± 5.55∗
5 *μ*g/mL licochalcone B	49.80 ± 4.91	48.27 ± 2.96	47.90 ± 4.25
CF			
Control	107.34 ± 3.16	107.09 ± 3.53	104.18 ± 5.97
I/R	90.57 ± 8.78^#^	100.72 ± 7.65^#^	99.92 ± 10.00^#^
0.5 *μ*g/mL licochalcone B	98.83 ± 1.78	98.72 ± 4.89	99.99 ± 3.45
1 *μ*g/mL licochalcone B	95.52 ± 6.18	97.95 ± 5.91	98.22 ± 7.16
3 *μ*g/mL licochalcone B	85.84 ± 3.91∗	90.73 ± 8.34∗	92.49 ± 7.77∗
5 *μ*g/mL licochalcone B	89.78 ± 5.44∗	90.07 ± 7.32∗	91.88 ± 3.67∗
HR			
Control	118.13 ± 8.05	118.96 ± 3.81	122.56 ± 3.91
I/R	86.49 ± 10.97^##^	78.49 ± 8.81^##^	69.00 ± 3.50^##^
0.5 *μ*g/mL licochalcone B	94.99 ± 8.90	93.01 ± 11.05∗	86.99 ± 1.34∗
1 *μ*g/mL licochalcone B	105.46 ± 8.29∗	100.55 ± 9.07∗∗	97.07 ± 4.36∗∗
3 *μ*g/mL licochalcone B	97.86 ± 8.53	89.68 ± 6.47	86.47 ± 5.02
5 *μ*g/mL licochalcone B	87.00 ± 9.40	80.49 ± 8.98	74.13 ± 3.57

Left ventricular developed pressure (LVDP); maximum rise velocity (+*dp*/*dt*_max⁡_); maximum down velocity (−*dp*/*dt*_max⁡_); coronary flow (CF); heart rate (HR). ^##^ *P* < 0.01 and ^#^ *P* < 0.05 compared with control group; *P < 0.05 and **P < 0.01 compared with the I/R group.

**Table 2:** Effect of licochalcone B on levels of CK and LDH in coronary flow of I/R injury (values are means with their standard deviation, *n* = 8).

Physical index	Before ischemia	Reperfusion	
	20 min	20 min	45 min
LDH (U/L)			
Control	18.3 ± 4.67	17.1 ± 3.84	16.70 ± 7.17
I/R	17.6 ± 6.37	64.0 ± 4.67^##^	58.5 ± 8.43^##^
0.5 *μ*g/mL licochalcone B	17.79 ± 5.14	37.53 ± 6.61∗∗	29.17 ± 5.61∗∗
1 *μ*g/mL licochalcone B	17.6 ± 5.64	22.5 ± 6.49∗∗	27.5 ± 7.26∗∗
3 *μ*g/mL licochalcone B	17.60 ± 7.70	55.38 ± 2.75∗	50.99 ± 3.42∗
5 *μ*g/mL licochalcone B	19.35 ± 7.91	60.51 ± 8.54	53.34 ± 5.50
CK (U/L)			
Control	28.19 ± 9.07	25.22 ± 5.89	26.02 ± 9.01
I/R	23.36 ± 7.55	366.98 ± 15.24^##^	126.36 ± 14.13^##^
0.5 *μ*g/mL licochalcone B	23.91 ± 3.95	276.47 ± 14.09∗∗	93.54 ± 17.35∗∗
1 *μ*g/mL licochalcone B	19.03 ± 11.12	243.63 ± 16.35∗∗	72.00 ± 17.24∗∗
3 *μ*g/mL licochalcone B	25.77 ± 9.73	310.97 ± 18.10	98.97 ± 14.89∗∗
5 *μ*g/mL licochalcone B	27.49 ± 5.88	325.17 ± 18.33	106.27 ± 18.77

^##^ *P* < 0.01 compared with the control group; *P < 0.05 and **P < 0.01 compared with I/R group.
